# Comparison between Single Dose Azithromycin and Six Doses, 3 Day Norfloxacin for Treatment of Cholera in Adult

**Published:** 2014-12

**Authors:** M. K. Bhattacharya, S Kanungo, T Ramamurthy, K Rajendran, A Sinha, A Bhattacharya, B. Sharma Sarkar

**Affiliations:** 1 National Institute of Cholera & Enteric Disease, Kolkata, India;; 2 ID & BG Hospital, Kolkata, India;; 3 Bankura Sammilani Medical College, India

**Keywords:** Single dose Azithromycin, Standard Norfloxacin, Adult cholera

## Abstract

**Aim:**

To evaluate the efficacy of single dose Azithromycin (1 gram) in treatment of cholera in adults. A randomized, controlled clinical trial on 120 adults with acute watery diarrhoea and moderate to severe dehydration compared the efficacy of azithromycin (1 gram) single dose and Norfloxacin (400 mg) twice daily for three days in treating cholera. Data were analysed for 64 patients who were stool culture positive for Vibrio cholerae. In conjunction with rehydration therapy, 32 patients received Azithromycin and 32 patients received Norfloxacin. Patients in the two treatment groups had comparable clinical characteristics on admission.

**Conclusions:**

Result shows Azithromycin and Norfloxacin has got almost similar efficacy in reducing stool output, duration of diarrhoea and fluid requirement in cholera positive cases.

## INTRODUCTION

Acute watery diarrhoea caused by V. Cholerae is an important cause of hospitalisation in Kolkata, India (1). Antibiotic therapy is a useful adjunct to fluid replacement in the treatment of cholera by substantially reducing the duration and volume of diarrhoea and thereby lessening fluid requirements and shortening the duration of hospitalization (2). These benefits are especially important in resource-constrained settings in which intravenous fluids, skilled care, and hospital beds may be in short supply, especially during cholera epidemics (3). Several drugs, namely tetracycline (4, 5), furazolidone (6), and trimethoprim-sulfamethoxazole (TMP-SMX) (7), have been

Found to be effective in reducing stool volume, duration of diarrhoea and vibrio excretion of the patient of cholera. But resistance towards different antibiotics have been reported from different parts of India. At present V. Cholerae showing significant resistance towards furazolidone and co-trimoxazole (8). Moreover tetracycline which is effective in single dose in treatment of cholera is also showing resistance (9, 10).

At present Norfloxacilin is showing good sensitivity against V. Cholerae (11) and it is being widely used for treatment of cholera. Unlike tetracycline it is not effective in single dose in treatment of cholera and it requires twice daily treatment for three days. Several studies have shown that a new macrolide, Azithromycin is highly active against common diarrhoea causing enteropathogen (12) and can be given in single dose (3).

In this study, we evaluated the comparative efficacy between single-dose Azithromycin (1 gram) and Norfloxacilin (400 mg) twice daily for three days, for the treatment of cholera in adults.

## MATERIALS AND METHODS

The study was conducted at Infectious Diseases Hospital, Kolkata, India from October 2010 to February 2012. It was a randomized controlled clinical trial. Male patients aged between 12 to 55 years( for ease of collection of stool and urine separately) with a history of acute watery diarrhoea of less than 24 hours durations with moderate to severe dehydration were included in the study. Female patients, patients having history of diarrhoea of more than 24 hours, patients with history of intake of antibiotics before hospitalization and any systemic illness e.g, pneumonia, meningitis were excluded from the study.

Written valid consent are taken from those patients (or from their guardian whatever is applicable) who fulfilled the inclusion criteria and had no exclusion criteria and were randomly assigned to one of the two treatment groups according to random number tables. One group received Azithromycin (1 gram) single dose and another group received Norfloxacin (400 mg) twice daily for three days. The randomization lists are prepared by a trained responsible person who was not involved in the study.

Stools were collected in sterile bottles and sent to laboratory in sterile container for culture tor identification of V. Cholerae on admission.

Patients were rehydrated with intravenous Ringer’s lactate solution and oral rehydration solution (ORS) according to WHO guidelines. Intake of fluids and output of stool and urine were recorded every 6h until diarrhoea stopped. The study was cleared by institutional ethics and scientific advisory committees.

### Statistical Analysis

The data were analysed by using SPSSPC 11.5 statistical package. Primary outcome variables ware total stool output and duration of diarrhoea and secondary outcome variables were total fluid intake (intravenous+ ORS).

## RESULTS

In total 120 adult patients 12-55 years age groups ware enrolled in the study with 60 patients in each treatment group. Of these 64 patients were positive for V. Cholerae infection, and 56 patients (28 patients from each treatment group) were negative for V. Cholerae. Of these 64 Cholera positive patients 32 received Azithromycin and 32 received Norfloxacin.

On admission characteristics such as age, body weight, frequency of diarrhoea, and preadmission duration of diarrhoea were comparables between the groups (Table 1).

**Table 1:** Admission characteristics and responses to the therapy of the two treatment groups

Variables	Azithromycin (n=32)	Norfloxacin (n=32)

**On admission**		
1. Age (y)	32.50 ± **12.06**	30.19 ± 10.88
2. Body weight (Kg)	50.59 ± 9.47	49.09 ± 8.20
**Pre admission**		
1. Duration of Diarrhoea (h)	10.36 ± 5.75	11.29 ± 6.02
2. Frequency of Stool ( times/day)	11.56 ± 2.97	10.28 ± 3.46
3. Frequency of Vomiting (times/day)	4.22 ± 2.35	3.75 ± **1.98**
**Responses to therapy**		
1. Total stool output (*ml*)	2518.75 ± 1498.21	2934.38 ± 2626.74
2. Total ORS intake (*ml*)	3965.63 ± 1765.75	3963.13 ± 1974.26
3. Total IV fluid intake (*ml*)	4565.63 ± 1316.06	5123.75 ± 1655.03
4. ORS+IV Fluid (ml)	8531.25 ± 2403.25	9086.88 ± 3237.91
5. Total duration of diarrhoea after treatment (h)	26.25 ± 7.67	29.75 ± 8.68
6. Total Urine output (ml)	1362.50 ± 487.75	1564.06 ± 841.30

Data are shown as mean ± standard deviation. ORS, oral rehydration solution.

Table 1 shows the major outcome variables in response to therapy. Difference between total stool output (2518.75 ml ± 1498.21 ml vs. 2934.38 ml ± 2626.74 ml) and total duration of diarrhoea after starting treatment (26.25h ± 7.67 h vs. 29.75h ± 8.68 h) between two treatment groups were statistically insignificant (*p*≥0.05). Moreover total fluid requirement ORS (3965.63 ml ± 1765.75 ml vs 3963.13 ml ± 1974.26 ml) and IV fluid (4565.63 ml ± 1316.06 ml vs. 5123.75 ml ± 1655.03 ml). The difference between total urine output (1362.50 ml ± 487.75 ml vs. 1564.06 ml ± 841.30 ml) and the difference between total ORS+IV Fluid is (8531.25 ml ± 2403.25 ml vs. 9086.88 ml ± 3237.91 ml) is comparable between two treatment groups.

## DISCUSSION

The problem of antimicrobial resistance in microorganisms causing cholera in both developed and developing countries continues to be alarming (13). Fluoroquinolones have excellent activity against all pathogenic *Vibrio* species, and clinical trials have found norfloxacin to be effective for the treatment of cholera in adults and in children (11, 14) and it is widely used in treatment of cholera. But some study reports are showing emergence of fluoroquinolone-resistant (norfloxacin) *V. cholerae* in India (15). However Azithromycin produced synthetically from Erythromycin by replacing 9a carbonyl in the glycone ring with methyl substituted nitrogen (16) has excellent sensitivity against V. Cholerae and no resistance emerged till now. Azithromycin has got good stability at low PH of stomach and its oral bioavailability is 37% which is almost similar to the bioavailability of norfloxacin(30-40%), however absorption is reduced by taking it with food or antacid (17).The serum half life is prolonged (2-3 days) allowing a once-a-cay dose regimen (18). These properties results in a shorter duration of therapy. The drug is rapidly delivered from reservoir to infected tissues and is eliminated in the stool without significant metabolism. The primary route of elimination is transintestinal and biliary, resulting in high concentration of drug in the stool (18).The present study also shows both groups are having comparable results in stool output duration of diarrhoea and fluid requirement( ORS+ IV fluid).

In conclusion Azithromycin appeared to be equal in efficacy to norfloxacin and may be regarded superior to norfloxacin because of its single dose treatment advantage. This single dose efficacy is quite helpful in cholera epidemics and may be used as a mainline drug in case of emergence of V. Cholerae resistant to Norfloxacin.
